# Successful Treatment of Stent Knot in the Proximal Ureter Using Ureteroscopy and Holmium Laser

**DOI:** 10.1155/2011/502191

**Published:** 2011-05-10

**Authors:** Masters M. Richards, Daniel Khalil, Ayman Mahdy

**Affiliations:** ^1^Department of Urology, University of Arkansas for Medical Sciences (UAMS), Little Rock, AR 72205, USA; ^2^Urology Division, Department of Surgery, University of Cincinnati (UC), Cincinnati, OH 45221, USA

## Abstract

Knotted ureteral stent is rare yet tedious complication that might represent a treatment challenge to the endourologist. Only twelve cases of knotted stent have been reported. Different management options have been reported, including simple traction, ureteroscopy, percutaneous removal, and open surgery. In this paper, we present the successful untying of the knot using ureteroscopy with holmium laser.

## 1. Introduction

Ureteral stents have been commonly used in urological procedures for more than three decades. Indications for placing stents include relief of ureteral obstruction, urinary diversion, and postoperative drainage [1]. As ureteral stenting has become more common, complications associated with stent insertion have also increased in frequency. One of the infrequently reported complications is the development of stent knots inside the ureter. There are only twelve cases of stent knotting reported in the literature. Management of this complication in those studies varied with simple traction succeeded in six, three days of continuous staged traction in one, percutaneous removal in two, untying the knot by grasping the upper end of the stent using ureteroscopy in one, untying the knot using amplatz superstiff guide wire in one and open surgery in one [2].

The Holmium laser is increasingly being used in the field of endourology. Its precision and safety allow for treatment of conditions throughout the genitourinary tract, including urinary calculi, strictures, prostate disease, soft tissue disorders, and foreign bodies. Holmium laser was successfully used in removal of weed trimmer and a detached resectoscope sheath tip in two different patients [3, 4]. 

In this study, we report the use of ureteroscopy with Holmium laser in treating a case of a retained double J stent that coiled around itself, forming a knot in the proximal ureter.

## 2. Case Presentation

**Figure 1:**
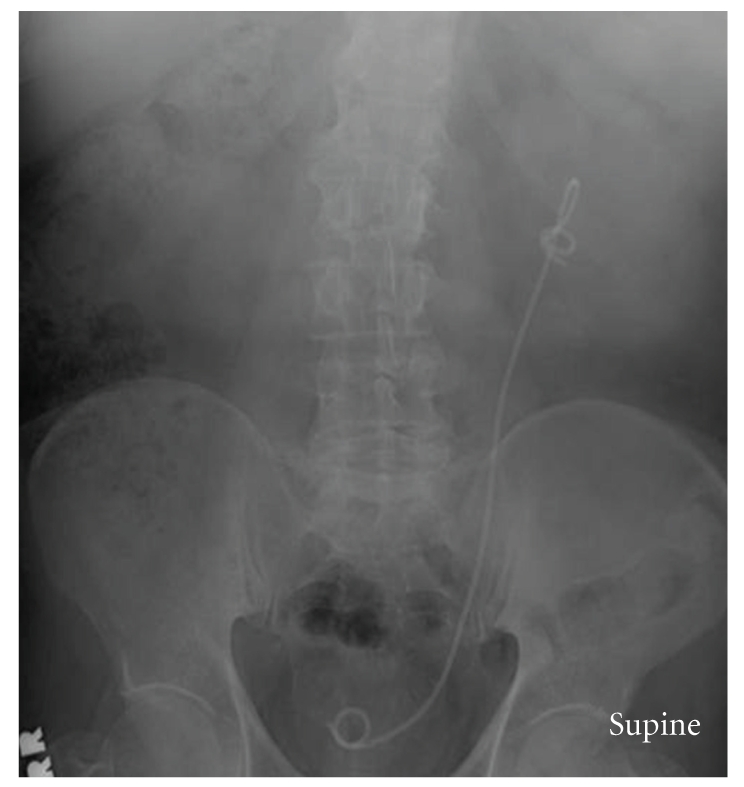
A plain X-Ray film shows the coiled proximal part of the JJ stent with formation of an incomplete knot.

A 67-year-old white male presented to the urology clinic stent removal. The patient had the stent inserted one month earlier following left ureteroscopy and stone retrieval. The stone was 7 mm in the proximal ureter. The stent was placed under direct cystoscopic and fluoroscopic guidance; it achieved adequate curling at the end of the procedure. Upon reviewing KUB films during followup, the proximal end of the stent was found to become coiled, creating a knot (Figure 1). Trial of the stent removal in the office failed with tightening of the knot during the attempted extraction. 

The decision was made to remove the stent under general anesthesia using the ureteroscope and Holmium laser to “undo” the knot. Using the 23-French cystoscope sheath, the scope was advanced into the bladder. The distal tip of the stent was grasped with the flexible grasper and gently extracted to the external urethral meatus and held with a hemostat. This was to provide counter traction during the lasing process and to help extraction as soon as the knot gets relieved. No excessive tension was applied at this point. The cystoscope was reinserted beside the stent to the bladder. A double floppy guide wire was advanced beside the double J stent up to the kidney. The bladder was emptied, and the cystoscope was removed. The ureteroscope was advanced into the left ureter until the proximal end of the stent was seen with a well-formed knot. At this point, the 365 holmium laser fiber was introduced into the scope, and the tip of the laser fiber was put in direct contact with the knot. The laser power used was 0.8 Joules and with frequency was 10 Hz. 

**Figure 2:**
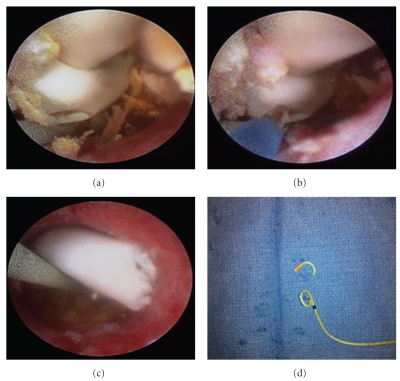
Summarizes the stages of the procedure; (a) The completely tightened stent knot as visualized during ureteroscopy, (b) The tip of the 365 um laser fiber directed to the knot, (c) The residual proximal fragment after extraction of the stent, (d) The two stent fragments after complete extraction.

The stent was extracted and the residual proximal fragment was grasped and removed (Figures 2(a)–2(d)). To our knowledge, this is the first reported use of Holmium laser in treating such a complication.

## 3. Discussion

Stent-related complications are well known and frequently reported problems in the field of endourology. These complications may include dysuria, flank pain, vesicoureteral reflux, urinary tract infection or obstruction, stent migration, stent encrustation, and stent fragmentation [1]. One of the less commonly reported complications is coiling of the stent with subsequent development of a knot during trial of extraction. If this occurs, stent knotting inside the ureter represents a treatment challenge. Excessive tension on the stent during extraction while the knot is impacted carries the risk of ureteral avulsion or stent cutting. 

Upon consulting the literature, several different treatment modalities have been utilized for treatment and removal of knotted double-J stents. The most common method is simple traction [2]. While trying to apply simple traction in our case, we encountered resistance and the knot become even tighter. This makes other reported conservative options for knotted stent removal difficult and invalid. 

The holmium laser has been widely used in the urology field with laser lithotripsy and prostate resection being the most common [5, 6]. The ability of holmium laser to fragment other foreign materials both in vitro and in vivo has been also reported [7]. The ability of holmium laser to fragment different objects is explained by its photothermal effect. This occurs when the treated material absorbs the laser energy and melts under the effect of the generated heat [8]. The holmium laser photothermal effect was also previously proved by Vassar et al. in an experimental study [9]. Using this data altogether and before we pursue more invasive options of percutaneous removal or open surgery, we thought of using the laser to break the center of the knot and remove the stent in two pieces. The procedure is simple and minimally invasive with no associated intra- or postoperative complications. 

While reporting this rare stent-related complication, we encourage decreasing the rate of stent use. In this case, stenting was indicated because of the relatively large size and proximal location of the stone in the ureter. In our practice we tend to avoid the routine ureteral stenting after noncomplicated ureteroscopy procedures. This approach is also supported by others [10, 11].

## 4. Conclusion

Stent knot is a rare complication but might be difficult to manage. If simple traction fails, the use of holmium laser in fragmenting and undoing the knot is an appropriate alternative. Selection of the correct length of stent and assuring good position and curle during insertion help avoid this complication.
